# Genetic vulnerability in patients with psychiatric presentations: a neuroscience perspective

**DOI:** 10.3325/cmj.2014.55.545

**Published:** 2014-10

**Authors:** Lukasz M. Konopka

**Affiliations:** Department of Psychiatry, Loyola Medical Center, Maywood Il, USA and Yellowbrick Consultation and Treatment Center, Evanston IL, USA

Today’s psychiatry is progressively entering the realm of science. Significant advances in structural neuroimaging and insight in functional as well as dysfunctional connectivity permit us to see the world from a patient’s perspective and generate hypotheses driven by objective brain-based data (1). In addition to these sophisticated imaging tools, human genetic mapping allows us to address an individual’s genetic vulnerabilities in relation to their brain’s development and processing internal and external information. Increasingly, psychiatric clinicians see genetic mapping as a valid and significantly beneficial tool (2). From a simple cheek swab or saliva sample, commercial enterprises give clinicians quick and easy access to a patient’s genetic vulnerabilities, which can be divided in two domains.

The first domain is related to psychotropic drug metabolism: If we know how well a patient’s liver enzymes deactivate and eliminate medications, we can choose a medication whose metabolism is neither too quick nor too slow. We must clearly understand both metabolism scenarios before prescribing any psychotropic medication because if the patient metabolizes a medication quickly, the medication may never achieve sufficient plasma levels to influence the brain. On the other hand, when an individual has deficient enzymatic machinery for a particular medication, they may develop significantly elevated drug concentrations that result in side effects and/or toxicity. In both cases, the medication may be deemed ineffective without truly assessing its efficacy, and the patient would have no benefit from the therapy (3). Moreover, a patient’s other medications may further interfere by activating or inhibiting the relevant enzymes. Thankfully, psychiatric therapeutic drug monitoring is emerging to address this issue (4).

Second, different genetically controlled biochemical targets influence therapeutic outcomes. Among these are the pre- and postsynaptic receptors, neurotransmitter transporter molecules, and voltage-gated ion channels. Of recent interest are enzymes involved in the degradation of released neurotransmitters. One such enzyme is catechol-O-methyltransferase (COMT), an enzyme that degrades dopamine and other catecholamines. This enzyme is present in two variants due to the single nucleotide polymorphism, resulting in a valine-to-methionine substitution at codon 158 (*COMT Val158Met*) (5). The patients who have the overactive enzyme are homozygous for valine form (*Val/Val*), which renders COMT hyper-efficient and may result in dopamine deficiency. In contrast, genotype *Met/Met* results in a hypo-active enzyme and causes excessive dopamine in the synaptic cleft. There are also heterozygotes *Val/Met*, carrying both variants of the enzyme. They are associated with intermediate function and often considered within the normal spectrum.

For now, let us focus on studies that investigate the consequences of hyper- and hypoactive COMT and ask whether individuals with more efficient COMT differ from the heterozygous population and from patients with less enzyme efficiency. For example, one study showed that Cannabis use may have significant negative effects in some vulnerable individuals and lead to psychosis and/or other chronic psychotic symptoms. It seemed that individuals who were homozygotes with *COMT Val/Val* alleles were more likely to develop and exhibit psychotic symptoms and could eventually develop schizophrenic-type disorders (6). This association was not observed in individuals whose COMT contained the Met substitution, an enzyme less effective in dopaminergic degradation (7). It also appeared that the COMT enzyme activity impacted brain function differently in men and women and gave them different predispositions to obsessive-compulsive type disorders (8). Individuals who were homozygotes for *Met* variant (*Met/Met*) took greater sexual risks than individuals with *Val/Val* genotype. Perhaps, excessive prefrontal dopaminergic activity causes cognitive dysfunction that, in turn, causes inappropriate decisions (9). A recent neuroimaging study asked whether *Val/Val* individuals, who had significantly more postsynaptic dopamine receptors, had lower dopamine levels as a consequence of pre-synaptic deprivation. The study confirmed the up-regulation of the cortical dopamine-1 receptor type in patients who were homozygous *Val/Val* compared to those who were homozygous *Met/Met*. The study evaluated the brain’s decision-making regions related to immediate and delayed gratification in COMT genotype patients. This study showed that individuals who carried the less active *COMT Met* variant exhibited a decreased immediate reward bias, while *Val/Val* individuals showed greater potential for impulsivity (10). Therefore, it may be that patients with *Val/Val* have significant processing insufficiency in frontal lobe circuits. Nevertheless, for most of these correlational studies, the hypotheses await additional studies to see if they will endure the test of time.

Finally, for psychopharmacology, genetic differences may have significant implications for how we choose medication for patients with specific genetic characterizations: Individuals with *Val/Val* gentoype have decreased frontal dopaminergic activity and potential executive function impairment. As such, agents such as stimulants may be considered for augmenting dopamine. Also, these individuals may respond to non-pharmacological prefrontal stimulations such as transcranial magnetic stimulation that seemingly increases dopamine activity (11,12). On the other hand, individuals who carry *COMT Met/Met* genotype have increased prefrontal dopamine and, thus, well-developed executive function that would respond negatively to stimulant therapy. How exciting to think that, until very recently, these genetic tools were simply fantasy. Now, these genetic developments have brought the world of mental health closer to the reality of individualized person-centered therapy.
